# Engineering Genetically Encoded Nanosensors for Real-Time *In Vivo* Measurements of Citrate Concentrations

**DOI:** 10.1371/journal.pone.0028245

**Published:** 2011-12-02

**Authors:** Jennifer C. Ewald, Sabrina Reich, Stephan Baumann, Wolf B. Frommer, Nicola Zamboni

**Affiliations:** 1 Institute of Molecular Systems Biology, ETH Zurich, Zurich, Switzerland; 2 PhD Program in Systems Biology of Complex Diseases, ETH Zurich, Zurich, Switzerland; 3 Department of Plant Biology, Carnegie Institution for Science, Stanford, California, United States of America; Cardiff University, United Kingdom

## Abstract

Citrate is an intermediate in catabolic as well as biosynthetic pathways and is an important regulatory molecule in the control of glycolysis and lipid metabolism. Mass spectrometric and NMR based metabolomics allow measuring citrate concentrations, but only with limited spatial and temporal resolution. Methods are so far lacking to monitor citrate levels in real-time *in-vivo*. Here, we present a series of genetically encoded citrate sensors based on Förster resonance energy transfer (FRET). We screened databases for citrate-binding proteins and tested three candidates *in vitro*. The citrate binding domain of the *Klebsiella pneumoniae* histidine sensor kinase CitA, inserted between the FRET pair Venus/CFP, yielded a sensor highly specific for citrate. We optimized the peptide linkers to achieve maximal FRET change upon citrate binding. By modifying residues in the citrate binding pocket, we were able to construct seven sensors with different affinities spanning a concentration range of three orders of magnitude without losing specificity. In a first *in vivo* application we show that *E. coli* maintains the capacity to take up glucose or acetate within seconds even after long-term starvation.

## Introduction

Metabolite concentrations are a functional read out of the state of metabolism [1]. In recent years, studies have exploited mass spectrometry or nuclear magnetic resonance based metabolomics approaches to elucidate gene functions and regulation in microbes and higher cells [2], [3], [4]. However, available metabolomics techniques have a limited temporal and spatial resolution due to disruptive sample preparation and the required sample amount, resprectively. Therefore, techniques allowing *in vivo* monitoring of metabolites are a valuable contribution to studying metabolism.

Fluorescent indicator proteins (FLIPs) have been developed to monitor metabolites *in vivo*. These genetically encoded sensors employ the concept of Förster resonance energy transfer (FRET) [5] to emit a fluorescent signal dependent on a ligand concentration. FLIPs consist of a protein which specifically binds a ligand and is sandwiched between two variants of green fluorescent protein (typically CFP and YFP). The efficiency of fluorescence energy transfer between the two fluorophores is highly dependent on their distance and orientation (reviewed in [6], [7], [8]). If the binding of a ligand induces a conformational change in the protein and alters the distance between the fluorophores, this is reflected in a change in the FRET efficiency.

Miyawaki et al. [9] firstly demonstrated this technique by measuring intracellular calcium during signalling events with the help of a FRET sensor based on calmodulin. Fehr et al. [10] presented the first FRET sensor for an organic molecule in 2002. Exploiting a periplasmic binding protein, they were able to develop a sensor for maltose and visualize its uptake in single yeast cells by fluorescence microscopy. To date, sensor proteins have been developed for approximately a dozen compounds and applied in various organisms including COS cells [11], Arabidopsis [12] , yeast [10], [13] and *Escherichia coli* [14]. Metabolite sensors have been developed thus far for signalling intermediates [15], [16], nucleotides [17], sugars [10], [11] and amino acids [18] (recently reviewed in [8]). Their main application has been to monitor exchange between cells and their environment. In this work we set out to develop a FRET sensor which allows monitoring of events downstream in intracellular carbon metabolism.

Citrate is an organic acid at the heart of central carbon metabolism. Its production is the first committed step of the tricarboxylic acid (TCA) cycle. This molecule is not only involved in catabolic processes, but is also a biosynthetic precursor and exhibits regulatory functions. It serves as substrate and as regulator for fatty acid synthesis [19], [20], which is of core interest in biomedical research. Citrate also regulates the glycolytic/gluconeogenic switch by allosteric control of the enzyme phosphofructokinase [21]. In eukaryotic cells the different functions of citrate take place in different compartments. Hence, the analysis of citrate dynamics calls for resolving concentrations in different compartments of the cell, which to date can only be achieved by genetically encoded sensors.

Here, we developed a series of FRET-based nanosensors for citrate. In a first *in vivo* application we monitor the dynamic responses of *E. coli* to addition of different carbon sources and show that cells maintain the capacity to immediately take up glucose and acetate even after 24 h of starvation.

## Results

### Candidate sensors

**Figure 1:**
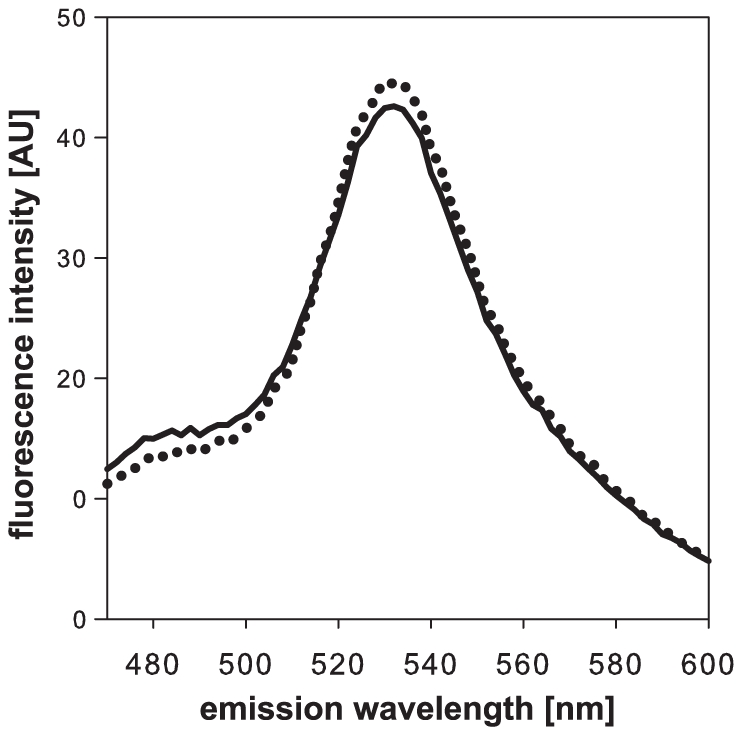
Fluorescence emission spectrum of CitA with and without 500 µM citrate. The FRET sensor CFP-CitA-Venus was purified and the fluorescence emission recorded at an excitation of 433 nm in a fluorescence plate reader. The dotted line represents the spectrum of the protein in buffer, the dashed line the protein after addition of 500 µM citrate.

We aimed at developing a FRET sensor for *in vivo*, real-time monitoring of citrate. Though several metabolite sensors have been constructed the design of novel sensors remains empirical. The key characteristics of a useful sensor are specificity and an affinity for the ligand in a physiological range. Since citric acid is only taken up and metabolized by a limited amount of organisms, natural citrate binding proteins were scarcer than for e.g. sugars or amino acids. We found three promising candidates: CitX (periplasmic binding protein from *Salmonella typhimurium*) and the binding domains of DpiB (a sensor histidine kinase from *E. coli*) and CitA (a sensor histidine kinase from *Klebsiella pneumoniae*). We cloned each of the candidate recognition elements between CFP and Venus (modified YFP [22]), inserting the sensors proteins directly between the fluorophores without additional linkers. We expressed the constructs in *E. coli* BL21 and analyzed the purified proteins *in vitro*. We recorded the fluorescence spectrum at an excitation of 433 nm and determined the ratio of the Venus (530 nm) to CFP (488 nm) peaks. All three candidates showed energy transfer from CFP to Venus. However, only CitA gave a small but reproducible change in fluorescence upon addition of 500 µM citrate (Figure 1). This sensor displayed a 530/488 nm fluorescence intensity ratio of 3.6 which increased to 3.9 upon ligand binding, which is in a similar range as most other non-optimized metabolite FRET sensors [23].

### Optimization and characterization *in vitro*

This observed change is small and may therefore limit practical application for *in vivo* measurements, where more background fluorescence and interference, e.g. light scattering, is expected. We therefore set out to increase the total FRET change upon citrate binding. This depends on the transduction of a conformational change in the receptor upon ligand binding into a positional change of the fluorophores. The CitA domain is a 132 amino acid protein that has been structurally and genetically characterized [24], [25], [26] (pdb file 2J80). We designed several truncations of CitA to remove potentially flexible elements in the peripheral areas of the protein. By combining three C-terminal and five N-terminal truncations we created a total of 15 CitA derivatives. We purified the modified proteins and determined the Venus/CFP ratio with or without addition of 500 µM citrate. While some truncations decreased or completely abolished ligand-induced change of FRET ratio, others significantly improved the maximal emission ratio change (Figure S1). The biggest improvement was obtained by removing residues 1–5 and 131–132, which constitute the unstructured regions preceding and following α-helices, respectively. The resulting nanosensor displayed a change in FRET ratio of 2.3 (55% increase), which is more than sixfold enhancement compared to the original construct. Further reducing the protein and removing the first three helices (which are not directly involved in citrate binding) had an adverse effect.

**Figure 2:**
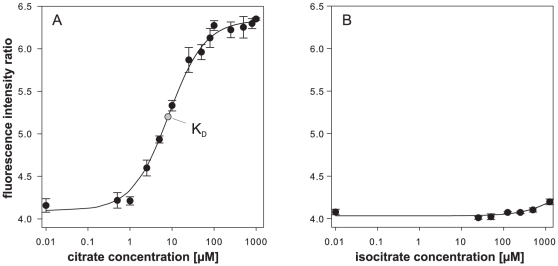
Binding curve of citrate to the FRET nanosensor CIT8μ. The FRET sensor CFP-truncated CitA-Venus was purified and the fluorescence emission of CFP and Venus recorded at an excitation of 433 nm in a fluorescence plate reader. The emission ratio 530/488 nm was determined at different citrate (A) and isocitrate (B) concentrations. Data points are averages of three independent protein extractions. Error bars indicate standard deviations. Data were fitted to a single site binding curve (black line) as described in the method section.

Next, we thoroughly tested the newly created nanosensor CFP-CitA6-130-Venus *in vitro*. We determined the binding properties by mixing the sensor with 0.5 to 1000 µM citrate. The obtained ratios were fitted to a single site binding curve as described in the methods section. We determined the K_D_, the ligand concentration at half-maximal saturation, to be 8 µM, which is very close to the value determined by Gerharz et al (2003) using isothermal titration calorimetry to characterize the purified protein [25]. The reproducibility between different batches of proteins extraction was excellent, with ratios in general not differing by more than 5%. A crucial feature for *in vivo* application is specificity. We thus exposed the nanosensor to a variety of structurally related organic acids including isocitrate, aconitate, α-ketoglutarate, succinate and pyruvate in presence or absence of citrate. With the exception of isocitrate, none of the tested compounds induced a detectable change in FRET ratio. In the case of isocitrate, we observed an effect only at concentrations above 1 mM (Figure 2 B) in absence of citrate, which by far exceeds the concentration typically observed by metabolomics in microbes [3]. Also, the apparent affinity for citrate was not modified in the presence of isocitrate or aconitate (data not shown), which excludes competitive binding. After confirming the high specificity of the sensor we tested pH stability, which is crucial when aiming to study certain intracellular compartments (Figure S2 A). An increase of pH only marginally affected the sensor and the binding curve at pH 7.5 was almost identical to pH 7. At pH 6.5 the FRET efficiency (Venus/CFP ratio) was reduced to a ratio of 3.5 (−15%), though the dynamic range and affinity were not affected. A further decrease of pH severely reduced FRET efficiency. Therefore, the new nanosensor should be functional in most compartments, however it is not suitable for highly acidic compartments such as endosomes or vacuoles [27]. We also tested the effect of high salt concentrations as typically observed in cells. Addition of 100 mM sodium chloride led to a major decrease of the FRET efficiency (Figure S2 B). Thus, as has been previously observed when comparing nanosensors *in vitro* and *in vivo* [13], [23], we can expect the observed fluorescence intensity ratio *in vivo* to be lower than under assay conditions. The K_D_ and the dynamic range, however, were not altered, and thus relative changes in citrate concentrations can still be monitored *in vivo*.

### Engineering affinity

The natural receptor CitA is highly sensitive and detects low concentrations of citrate in the periplasmic environment and thus has a very high affinity for its substrate. For application as an intracellular sensor a saturation concentration of approximately 100 µM appeared too low given the estimates of intracellular concentrations of citrate by metabolomics approaches in the mid micromolar to low millimolar range [3]. We therefore engineered the affinity of the nanosensor for *in vivo* applications. Gerharz et al [25] systematically investigated residues of the CitA sensor domain involved in citrate binding. Replacement of positively charged amino acids with alanine led to decreased affinity. We chose five of the reported point mutations and introduced them into the sensor CitA6-130. We determined binding constants and total FRET ratios from two independent extractions (Table 1). The binding constants were qualitatively in good agreement with the observations of Gerharz et al. [25], though in some cases we observed a much larger decrease of affinity. This might be due to the different measurement techniques, a different behaviour of the truncated and sandwiched protein compared to the isolated full-length protein, or to the minor differences we found in the sequence compared to the published *Klebsiella* CitA gene (sequence of the final sensor can be found in Document S1). The mutation R66A completely abolished citrate-induced FRET change. The others increased the apparent K_D_ 2–250 fold up to 1.8 mM (R107A). To further increase the coverage of affinities we combined the two mutations K77A and R49A in one sensor and thereby obtained an affinity of 470 µM. In total, our set of six citrate nanosensors cover an affinity range of 8 µM to 1.8 mM, and a dynamic measurement range of 1 µM–15 mM (10–90% saturation). Decreasing the affinity for the designated substrate bears the risk of also reducing specificity. We therefore tested all mutated sensors for binding of isocitrate and aconitate, the most closely related compounds. All of the sensors maintained their specificity and showed no FRET change in response to addition of either compound. Also, pH and salt sensitivity were similar to the original sensor (data not shown).

**Table 1:** Binding properties of seven citrate sensors.

sensor	mutation	K_D_	R_apo_	R_sat_	ratio change
		[µM]			(in % R_apo_)
CIT8μ	truncation	8 µM	4.1	6.4	55
CIT50μ	R49A	50 µM	4.8	6.2	29
CIT0	R66A	no binding	4.3	4.3	0
CIT96μ	K77A	96 µM	4.1	6.4	55
CIT32μ	K92A	32 µM	4.5	6.5	44
CIT1.8m	R107A	1765 µM	4.4	5.3	20
CIT0.5m	R49A-K77A	470 µM	4.3	5.8	35

Values are averages of two independent protein extractions, measured twice each. R_apo_: Venus/CFP ratio at 0 µM citrate; K_D_: concentration at half-maximal saturation.

### Monitoring the dynamic response of *E. coli* to different substrates after starvation

**Figure 3:**
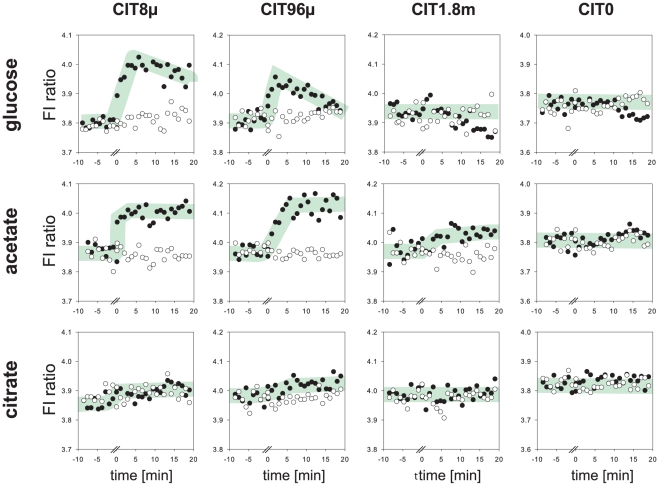
Response of citrate concentration in *E. coli* upon addition of different carbon substrates after starvation. The response was monitored by citrate nanosensors of different affinities. Time zero indicates the addition of glucose, acetate or citrate; this interrupted the measurement for approximately 20 s. White dots represent the buffer controls. FI: fluorescence intensity.

One of the major advantages of using FRET sensors is the ability to collect real time data to study the kinetics of metabolite accumulation. In a first *in vivo* application we monitored the metabolic response of *E. coli* to different substrates after starvation. After starving cells harbouring different versions of the citrate sensor in carbon-free M9 media for four hours we added glucose, acetate, citrate or buffer control to the cells and monitored their response over time. Time profiles are shown in Figure 3. We observed an immediate increase of the FRET ratio in bacteria supplied with either glucose or acetate, but not with citrate or the carbon-free control. The addition of substrate did not elicit a ratio change in the sensor CIT0, which does not bind citrate *in vitro*. This confirms that the observed effect is specific to a change in citrate concentration. The external supply of citrate did not lead to an increase of intracellular citrate concentration, consistent with the observation that *E. coli* does not metabolize citrate as a carbon source under aerobic conditions [28].

While in acetate medium citrate concentrations remain high, on glucose a transient peak is followed by a drop in concentration. This observation fits well to those made by Bennett et al [3], who show that citrate concentrations during growth on acetate exceeded the concentrations during growth on glucose more than fivefold. The initial citrate peak upon glucose uptake might be due to a higher glycolytic than TCA cycle capacity during starvation. Thus, citrate is accumulated until expression of the aconitate hydratase and downstream enzymes is induced. Whether the small differences in the speed of the glucose or acetate pulse to manifest in the citrate pool results from slower uptake or lower capacity of the downstream enzymes remains to be investigated.

The FRET ratios *in vivo* differ from those determined under assay conditions and therefore the ratio does not directly allow inferring the absolute concentrations. However, using sensors with different affinities allows roughly estimating the concentration range. Upon a glucose pulse, both the sensor CIT8μ and the sensor CIT96μ, but not the sensor CIT1.8m respond, indicating a concentration in the mid micromolar range. The initial ratio varied by about 0.1 around the midpoint, which could either be caused by variations in the base level of citrate or could be caused by other parameters such as optical density, sedimentation etc. It was thus not possible to directly compare steady state levels, however under optimized conditions FRET sensors can be used to monitor steady state levels as shown by Bermejo et al. [13].

**Figure 4:**
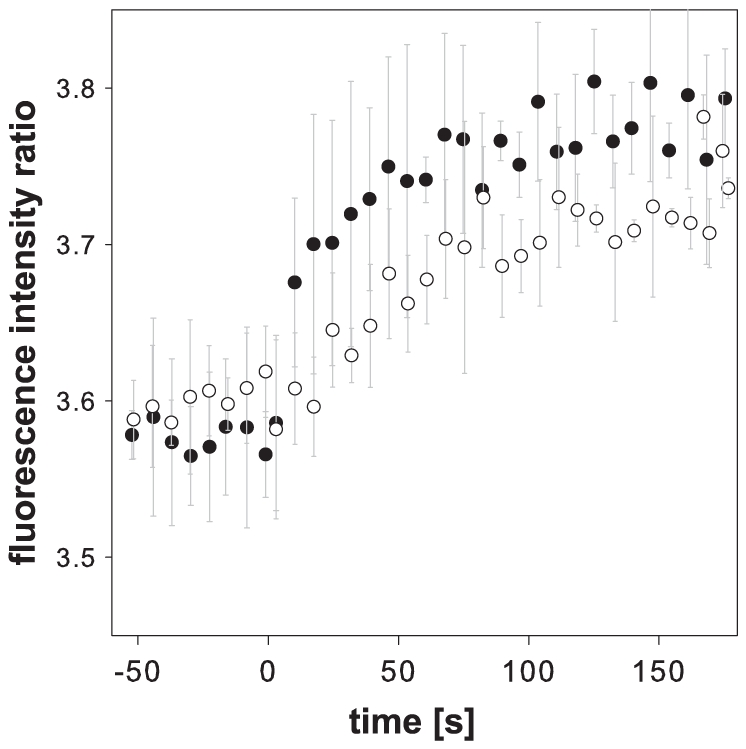
Citrate concentration changes in *E. coli* in response to carbon substrates after 24 hours of starvation. Addition of substrates is indicated as time point t = 0. Manual addition interrupted the measurement for approximately 10 s. Black dots: glucose, white dots: acetate. Time points are averaged over three independent experiments.

Next, we investigated whether *E.coli* maintains glucose and acetate uptake capacity even after an extended starvation periods of 24 hours. After 1 day in carbon-free M9 medium we subjected cells harbouring the FLIP-CIT8μ plasmid to a pulse of either glucose or acetate and monitored the intracellular response at a temporal resolution of approximately seven seconds (Figure 4). We observed a rapid response of the citrate level which reached a maximum already after 50 s and 90 s upon a glucose or acetate addition, respectively. Thus, even after a long period of complete carbon starvation *E. coli* is able to immediately take up and utilize common carbon substrates. The presence of glucose activity even in the absence of the substrate allows the cells to immediately acquire the preferred nutrient when it becomes available, similarly as observed in yeast [13].

## Discussion

Genetically encoded nanosensors allow minimally invasive, timely and spatially resolved monitoring of metabolites. While several FRET nanosensors have been developed for amino acids and sugars (reviewed in [29]), sensors to monitor events further downstream in metabolism were still lacking. In this work we constructed a fluorescence nanosensor for citrate. To the best of our knowledge, this is the first sensor for an organic acid. Using a structure-based approach to increase the rigidity of the protein we were able to enhance the total change in FRET six-fold, achieving a total ratiometric change of 2 (55% increase). By introducing point mutations we engineered a total of six sensor versions spanning three logs of affinities, and additionally generated a control sensor unable to bind citrate.

We constructed the first sensor utilizing the periplasmic domain of a sensor kinase. This successful example opens up a new toolbox of potential protein scaffolds for the construction of novel sensors. Histidine sensor kinases are part of the bacterial two component response systems and are widely spread in the bacterial world, sensing a great variety of substrates including different organic acids [30]. Similar to the typical hinge-bend movement observed in periplasmic binding proteins, periplasmic sensor domains of histidine kinases undergo strong conformational change to transduce a signal [31] and are therefore ideal candidates for FRET sensors.

As first proof of principle, we demonstrate the successful application of the sensor to monitor substrate shifts in *Escherichia coli*. Using this *in vivo* approach we were able to show that even after a long starvation period *E. coli* can take up and metabolize glucose through glycolysis within several seconds after glucose addition. Due to the difference in FRET efficiency between assay and *in vivo* conditions, the fluorescence ratio does not so far allow estimating absolute intracellular concentrations. However, combining read-outs from sensors with different affinities and cell-averaged concentrations obtained by by mass spectrometry could allow reasonable estimates.

This series of citrate sensors allows non-invasively monitoring citrate in microbes. Recently, FRET sensors have successfully been implemented in microbes for monitoring glucose steady state levels, the capacity of starved yeast cells to accumulate glucose, for the identification of the nature of the transport using mutant screens and for identifying novel sugar transporters [13], [32]. Our suite of citrate sensors could be employed to follow metabolic activity as demonstrated in this study, to elucidate potential regulatory functions of citrate or to facilitate metabolic engineering for citric acid production. This work also paves the road to elucidate compartment-specific dynamics and regulatory events in higher cells that involve or are mediated by citrate. It was recently shown that citrate is the precursor for histone acetylation in mammalian cells and thus links chromatin remodelling to energy metabolism [33]. A citrate sensor could further shed light on mechanisms and dynamics involved in this interesting regulatory circuit.

## Materials and Methods

### Construction of candidate sensors

Candidate sensors were amplified from genomic DNA of *Klebsiella pneumonia*e, *Salmonella typhimurium* and *Escherichia coli*, respectively, by polymerase chain reaction (PCR) using primers as specified in Table 2. The PCR product was inserted between the fluorophores in plasmid pGW1 [14] (based on pRSET) using restriction sites KpnI and SpeI.

**Table 2:** Primers used for construction and engineering of sensor proteins.

gene	primer including restriction sites
**CitX**	
***Salmonella typhimurium***	
forward	CCCCCCGGTACCATGAAGGGCACAGACCTGTTG
reverse	CCCCCCACTAGTGTCACAACGCTCACGTT C
**DpiB**	
***Eschericia coli***	
forward	CCCCCCGGTACCGCGCAATATTTTACGGCC AGT
reverse	CCCCCCACTAGTAGCCCGCCAGCTATCGATTTT
**CitA**	
***Klebsiella pneumoniae***	
forward	CCCCCCGGTACCATGGACATTACCGAGGAGCGTCTG
reverse	CCCCCCACTAGTCTCCAGTTGCTCGATGGTATAGCC
**truncated versions forward**	
amino acid 5	CCCCCCGGTACCGAGCGTCTGCATTATCAGGTCGGG
amino acid 6	CCCCCCGGTACCCGTCTGCATTATCAGGTCGGG
amino acid 7	CCCCCCGGTACCCTGCATTATCAGGTCGGGCAA
amino acid 13	CCCCCCGGTACCGGGCAACGGGCGCTGATTCAG
amino acid 51	CCCCCCGGTACCTCCGACGCCACCTACATCACC
**truncated versions reverse**	
amino acid 126	CCCCCCACTAGTGCCTACCGACACAATGCCGATCAC
amino acid 128	CCCCCCACTAGTGGTATAGCCTACCGACACAATGCC
amino acid 130	CCCCCCACTAGTTTGCTCGATGGTATAGCCTACCGA

### Construction of CitA variants

Truncations of the CitA protein were achieved by PCR- amplifying truncated forms from the original CFP-CitA-Venus construct (using primers listed in Table 2) and inserting them into pGW1 [14] using restriction sites KpnI and SpeI. The sequence of the final sensor FLIP-CIT8μ can be found in Document S1. Point mutations were introduced either with the QuickChange Kit (Stratagene) or by mutation and fusion PCR using the primers published in [25]. Mutations were verified by Sanger sequencing of both strands (Microsynth, Switzerland).

### Expression and purification of proteins

Protein expression and purification protocol was modified from ref. [10]. *E. coli* BL21 harboring the nanosensor-encoding plasmid were grownin LB medium (supplemented with 100 mg/l ampicillin), at room temperature for 72 h. Cells were harvested by centrifugation, washed twice in 20 mM Tris/HCl (pH 8) and then resuspended in the same buffer. Cells were disrupted by sonication and cell debris was removed by centrifugation. The supernatant was applied to a Ni+ column for His-tag purification (GE Healthcare, either His-Trap or Gravi-Trap) using 20 mM Tris/HCl (pH 8) for washing and 200 mM imidazol for elution of the proteins. To remove imidazol and extraction buffer, the protein solution was concentrated to 50 µl in a Vivaspin500 (5 kDa molecular cut-off, sartorius stedim biotech, Germany), washed with 500 µl assay buffer (20 mM MOPS, 20 mM NaCl) and finally diluted tenfold in assay buffer. Proteins were stored at 4°C overnight before analysis to allow proper folding. SDS PAGE indicated high purity as no additional polypeptides were detected.

### *In vitro* assays

All in vitro assays were performed in a TECAN Infinite 200 plate reader (TECAN, Austria) in 96 well format (modified from [10]). Proteins, reagents and ligands were dissolved in assay buffer (20 mM Mops, 20 mM NaCl, pH 7 unless specified otherwise). Typically, 100 µl total volume per well were used. Protein concentration was adjusted so that both Venus and CFP emission were in the linear range of the detector at emission gain 80. Twofold higher or lower protein concentrations did not affect the outcome of the assay. Emission wavelengths 488 nm and 530 nm, or a continuous spectrum from 470–580 nm, were monitored at an excitation of 433 nm. A blank measurement was obtained from a well containing only assay buffer and subtracted from samples.

Measured intensity ratios were fitted to a single site binding curve:
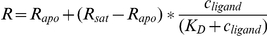



### *E. coli in vivo* experiment

*In vivo* experiments were adapted from [14] and [34]. *E. coli* BL21 cells were grown for 60 h in LB medium at room temperature. Cultures were stored over night at 4°C and then starved in carbon free M9 medium for 4 or 24 hours at 37°C. 190 µl cultures were transferred to 96 well plates. Fluorescence emission at 488 and 530 nm (excitation 433 nm, bandwidth 2 nm) were recorded in a TECAN Infinite 200 plate reader, shaking at 170 rpm between readings. 10 µl glucose, acetate, citrate (all final concentration 2 g/l) or carbon-free M9 were added manually to the cultures.

## Supporting Information

Figure S1 **FRET ratio of CitA variants.** Truncated variants of CitA were created as described in the methods section. The naming indicates the first and last amino acid. We determined the FRET ratio in buffer (black bars) and the change in ratio (i.e. Δ FRET) upon addition of 500 µM citrate (grey bars).
(TIF)

Figure S2 **The effect of pH and salt concentration on the sensor FLIP CIT8μ.** FRET ratios were measured for 7 citrate concentrations in duplicate and binding curves were fitted as described in the methods section. (A) citrate and proteins were prepared in MOPS-buffer at the pH indicated in the chart, (B) Binding curve was determined in MOPS buffer (pH 7) containing 20 mM (normal assay conditions) and 100 mM sodium chloride.
(TIF)

Document S1 Nucleotide sequence of the sensor FLIP CitA6-130.
(DOC)
