# Supplementary material for: Engineering Genetically Encoded Nanosensors for Real-Time In Vivo Measurements of Citrate Concentrations
Source: PLoS One. 2011 Dec 2;6(12):e28245. doi: 10.1371/journal.pone.0028245 (PMC3229521; doi:10.1371/journal.pone.0028245)
Supplement: Document S1 — Nucleotide sequence of the sensor FLIP CitA6-130. (DOC) [file pone.0028245.s003.doc]

**1 ATCTCGATCC CGCGAAATTA ATACGACTCA CTATAGGGAG ACCACAACGG**

**51 TTTCCCTCTA GATAATTTTG TTTAACTTTA AGAAGGAGAT ATACATATGC**

**101 GGGGTTCTCA TCATCATCAT CATCATGGTA TGGCTAGCAT GACTGGTGGA**

**151 CAGCAAATGG GTCGGGATCT GTACGACGAT GACGATAAGG ATCCGGGCCT**

**201 CGAGGTGAGC AAGGGCGAGG AGCTGTTCAC CGGGGTGGTG CCCATCCTGG**

**251 TCGAGCTGGA CGGCGACGTA AACGGCCACA AGTTCAGCGT GTCCGGCGAG**

**301 GGCGAGGGCG ATGCCACCTA CGGCAAGCTG ACCCTGAAGT TCATCTGCAC**

**351 CACCGGCAAG CTGCCCGTGC CCTGGCCCAC CCTCGTGACC ACCCTGACCT**

**401 GGGGCGTGCA GTGCTTCAGC CGCTACCCCG ACCACATGAA GCAGCACGAC**

**451 TTCTTCAAGT CCGCCATGCC CGAAGGCTAC GTCCAGGAGC GCACCATCTT**

**501 CTTCAAGGAC GACGGCAACT ACAAGACCCG CGCCGAGGTG AAGTTCGAGG**

**551 GCGACACCCT GGTGAACCGC ATCGAGCTGA AGGGCATCGA CTTCAAGGAG**

**601 GACGGCAACA TCCTGGGGCA CAAGCTGGAG TACAACTACA TCAGCCACAA**

**651 CGTCTATATC ACCGCCGACA AGCAGAAGAA CGGCATCAAG GCCAACTTCA**

**701 AGATCCGCCA CAACATCGAG GACGGCAGCG TGCAGCTCGC CGACCACTAC**

**751 CAGCAGAACA CCCCCATCGG CGACGGCCCC GTGCTGCTGC CCGACAACCA**

**801 CTACCTGAGC ACCCAGTCCG CCCTGAGCAA AGACCCCAAC GAGAAGCGCG**

**851 ATCACATGGT CCTGCTGGAG TTCGTGACCG CCGCCGGGGG TACCCGTCTG**

**901 CATTATCAGG TCGGGCAACG GGCGCTGATT CAGGCGATGC AGATTTCGGC**

**951 GATGCCGGAG CTGGTGGAGG CGGTACAGAA ACGCGACCTC GCCAGAATCA**

**1001 AAGCCCTTAT CGACCCCATG CGTTCGTTCT CCGACGCCAC CTACATCACC**

**1051 GTCGGTGATG CCAGCGGCCA GCGCCTCTAT CACGTCAATC CTGATGAAAT**

**1101 CGGCAAATCG ATGGAAGGCG GCGATAGCGA TGAGGCGTTG ATAAACGCCA**

**1151 AAAGCTACGT GTCAGTGCGC AAAGGCTCGC TGGGATCCTC GTTGCGTGGT**

**1201 AAATCGCCGA TCCAGGACGC GACCGGCAAG GTGATCGGCA TTGTGTCGGT**

**1251 AGGCTATACC ATCGAGCAAA CTAGTAAGGG CGAGGAGCTG TTCACCGGGG**

**1301 TGGTGCCCAT CCTGGTCGAG CTGGACGGCG ACGTAAACGG CCACAAGTTC**

**1351 AGCGTGTCCG GCGAGGGCGA GGGCGATGCC ACCTACGGCA AGCTGACCCT**

**1401 GAAGCTGATC TGCACCACCG GCAAGCTGCC CGTGCCCTGG CCCACCCTCG**

**1451 TGACCACCCT GGGCTACGGC CTGCAGTGCT TCGCCCGCTA CCCCGACCAC**

**1501 ATGAAGCAGC ACGACTTCTT CAAGTCCGCC ATGCCCGAAG GCTACGTCCA**

**1551 GGAGCGCACC ATCTTCTTCA AGGACGACGG CAACTACAAG ACCCGCGCCG**

**1601 AGGTGAAGTT CGAGGGCGAC ACCCTGGTGA ACCGCATCGA GCTGAAGGGC**

**1651 ATCGACTTCA AGGAGGACGG CAACATCCTG GGGCACAAGC TGGAGTACAA**

**1701 CTACAACAGC CACAACGTCT ATATCACCGC CGACAAGCAG AAGAACGGCA**

**1751 TCAAGGCCAA CTTCAAGATC CGCCACAACA TCGAGGACGG CCGCGTGCAG**

**1801 CTCGCCGACC ACTACCAGCA GAACACCCCC ATCGGCGACG GCCCCGTGCT**

**1851 GCTGCCCGAC AACCACTACC TGAGCTACCA GTCCGCCCTG AGCAAAGACC**

**1901 CCAACGAGAA GCGCGATCAC ATGGTCCTGC TGGAGTTCGT GACCGCCGCC**

**1951 GGGATCACTC TCGGCATGGA CGAGCTGTAC AAGTAA**
